# Growth of *Leptospirillum ferriphilum* in sulfur medium in co-culture with *Acidithiobacillus caldus*

**DOI:** 10.1007/s00792-018-1001-3

**Published:** 2018-01-12

**Authors:** Sarah L. Smith, D. Barrie Johnson

**Affiliations:** College of Natural Sciences, Bangor University, Deiniol Road, Bangor, LL57 2UW UK

**Keywords:** *Acidithiobacillus*, Acidophiles, Iron cycling, Iron reduction, *Leptospirillum*, Microbial interactions

## Abstract

*Leptospirillum ferriphilum* and *Acidithiobacillus caldus* are both thermotolerant acidophilic bacteria that frequently co-exist in natural and man-made environments, such as biomining sites. Both are aerobic chemolithotrophs; *L. ferriphilum* is known only to use ferrous iron as electron donor, while *A. caldus* can use zero-valent and reduced sulfur, and also hydrogen, as electron donors. It has recently been demonstrated that *A. caldus* reduces ferric iron to ferrous when grown aerobically on sulfur. Experiments were carried out which demonstrated that this allowed *L. ferriphilum* to be sustained for protracted periods in media containing very little soluble iron, implying that dynamic cycling of iron occurred in aerobic mixed cultures of these two bacteria. In contrast, numbers of viable *L. ferriphilum* rapidly declined in mixed cultures that did not contain sulfur. Data also indicated that growth of *A. caldus* was partially inhibited in the presence of *L. ferriphilum*. This was shown to be due to greater sensitivity of the sulfur-oxidizer to ferric than to ferrous iron, and to highly positive redox potentials, which are characteristic of cultures containing *Leptospirillum* spp. The implications of these results in the microbial ecology of extremely acidic environments and in commercial bioprocessing applications are discussed.

## Introduction

The ability to grow by the dissimilatory oxidation of inorganic electron donors (ferrous iron, hydrogen, sulfur and reduced inorganic sulfur anions) is widespread amongst acidophilic prokaryotes (Quatrini and Johnson 2016). Both oxygen and ferric iron can act as electron acceptors for many species of chemolithotrophic acidophiles, enabling them to exploit anoxic as well as aerobic environments. Some bacteria (e.g. *Acidithiobacillus ferridurans* and *Acidithiobacillus ferrooxidans*) can use all of the electron donors listed above, while other species (e.g. *Acidithiobacillus ferriphilus* and *Acidithiobacillus ferrivorans*) have more restricted metabolic capabilities. *Leptospirillum* (*L*.) spp. (*L. ferrooxidans, L. ferriphilum* and *L. ferrodiazotrophum)* have been shown to use only ferrous iron as electron donor and are therefore (as a result of thermo-dynamic constraints) obligate aerobes (Dopson 2016). The thermo-tolerant acidophile *Acidithiobacillus caldus* can grow by oxidizing reduced and elemental sulfur and, at least in the case of the type strain, molecular hydrogen (Hallberg and Lindstrom 1994; Hedrich and Johnson 2013). While all four currently recognised species of iron- and sulfur-oxidizing *Acidithiobacillus* (*A. ferridurans, A. ferrooxidans, A. ferrivorans* and *A. ferriphilus*) can grow in the absence of oxygen by ferric iron respiration, *A. caldus* and *Acidithiobacillus thiooxidans* (both sulfur-oxidizing acidophiles do not oxidize ferrous iron) cannot do so, even though cultures have been reported to reduce ferric iron (Brock and Gustafson 1976; Hallberg et al. 2001).

Sand (1989) found that aerobic cultures of *A. ferrooxidans* grown on elemental sulfur accumulated ferrous iron when culture pH decreased to 1.3, which is the lower pH limit for growth of this acidophile. More recently, Johnson et al. (2017) showed that the ability to reduce ferric iron to ferrous in aerated bioreactors and shake flasks was widespread among the acidithiobacilli. For those species that could also oxidize ferrous iron, reduction was not significant until the pH had fallen (due to production of sulfuric acid from the oxidation of sulfur) to ~ pH 1.3, while ferrous iron accumulated in aerobic cultures of *A. caldus* at higher pH values (at and below pH 2.0). It was also noted that similar rates of iron reduction occurred in the presence and absence of bacteria, suggesting that it was an indirect process mediated by extracellular metabolite(s). Cultures of *A. caldus* grown aerobically on hydrogen were also found to reduce ferric iron. This apparent indirect and passive reduction of iron mediated under aerobic conditions by acidophiles that lack the ability to re-oxidize the ferrous iron generated would, in theory, be beneficial to other species that can use energy associated with ferrous iron oxidation to sustain their growth. We have tested this hypothesis by growing mixed cultures of sulfur-oxidizing *A. caldus* and iron-oxidizing *L. ferriphilum* aerobically, with sulfur as the major electron donor provided.

## Materials and methods

### Bacteria and culture conditions

*A. caldus* (DSM 8584^T^) and *L. ferriphilum* strain MT63 (which had previously been isolated from a pilot-scale mineral bioleaching operation; Okibe et al. 2003) were used in experimental work. Both strains are maintained in the *Acidophile Culture Collection* at Bangor University (UK), and were grown routinely in shake flasks incubated at 45 °C. *A. caldus* was grown in a basal salts/trace elements medium (Nancucheo et al. 2016) supplemented with 0.5% (w/v) elemental sulfur (initial pH 2.0) while *L. ferriphilum* was grown in a basal salts/trace elements medium supplemented with 20 mM ferrous sulfate (initial pH 1.7).

### Growth of *A. caldus* and *L. ferriphilum* in pure and mixed cultures (experiment 1)

Culture media (100 or 500 mL in 250 mL or 1 L shake flasks) containing 0.5% zero valent sulfur (equivalent to 156 mM S^0^) and 5 mM ferrous iron and adjusted (with sulfuric acid) to pH 1.8, were prepared and inoculated with pure cultures of both *A. caldus* and *L. ferriphilum*. Two control cultures were prepared at the same time. Both of these were as described above, except that elemental sulfur was omitted from one while the other contained 5 mM ferric (rather than ferrous) sulfate and was inoculated with only *A. caldus*. The flasks were incubated at 45 °C, shaken at 100 rpm for up to 55 days. Small volumes of culture liquors were removed regularly and analyzed for pH, redox potentials (recorded as *E*_H_ values, i.e. corrected against a standard hydrogen electrode) ferrous and total iron, and total numbers of planktonic bacteria and viable *L. ferriphilum*. Sterile water was routinely added to the cultures to compensate for that lost by evaporation.

### Impact of oxidized medium of *L. ferriphilum* on growth and sulfur oxidation by *A. caldus* (experiment 2)

Data from the first experiment suggested that *L. ferriphilum* had a negative impact on *A. caldus* in terms of its growth and ability to oxidize elemental sulfur. To confirm this observation, a pure culture (450 mL) of *L. ferriphilum* was grown in a 1 L shake flask containing 20 mM ferrous iron, 2× basal salts/trace elements solution, and an initial pH of 1.5. Doubling the concentrations of the basal salts and trace elements ensured that any subsequent negative activity of *A. caldus* detected was not due to a deficiency of a macro- or micro-nutrient. The culture was grown aerobically (at 45 °C, with agitation) to the completion of iron oxidation, and pH, *E*_H_ and cell numbers recorded. The oxidized culture liquor was then split into two equal volumes. Bacteria were removed from one batch (by centrifuging at 3000×*g* for 20 min), followed by filtration through 0.2 μm membrane filters. One hundred millilitre aliquots of either cell-containing or “cell-free” oxidized culture liquors were then placed into four sterile 250 mL conical flasks and 500 mg of sterile elemental sulfur added to each. Control cultures containing 2× concentrations of basal salts and trace elements, 20 mM ferric iron (added from a stock solution of sterile ferric sulfate) and adjusted to the same pH as the oxidized *L. ferriphilum* cultures, were also set up. All six flasks were inoculated with an active culture of *A. caldus* and incubated aerobically at 45 °C for 13 days. Samples from the cultures were analyzed for pH, *E*_H_, cell numbers and concentrations of ferrous iron and sulfate.

### Comparative effects of ferrous and ferric iron on sulfur oxidation by *A. caldus* (experiment 3)

An experiment was set up to determine whether the partial inhibition of growth and sulfur oxidation by *A. caldus* observed in mixed cultures with *L. ferriphilum* was due to ferric iron generation by the latter. For this, liquid media containing 0.5% (w/v) zero valent sulfur and basal salts/trace elements were supplemented with 5 mM ferrous sulfate, or 5 or 25 mM ferric sulfate. These were inoculated with *A. caldus*, incubated (shaken at 45 °C) for up to 20 days, and analysed at regular intervals for pH, *E*_H_, sulfate concentrations and numbers of planktonic cells.

### Physico-chemical and microbial analyses

pH values were measured using a pHase glass combination electrode (VWR, UK) and redox potentials using a combination platinum/silver–silver chloride electrode (Thermo Scientific, UK). Both electrodes were coupled to an Accumet 50 pH meter. Ferrous iron concentrations were determined colorimetrically using the Ferrozine assay (Stookey 1970) and total soluble iron was determined by firstly reducing ferric iron to ferrous with an excess of ascorbic acid, and assaying again with Ferrozine. Concentrations of sulfate were determined by ion chromatography using a Dionex C25 ion chromatograph with an Ion Pac AS-11 column fitted with a conductivity detector. Total planktonic bacteria were enumerated using a Thoma counting chamber using a phase contrast microscope at 400× magnification. Numbers of viable *L. ferriphilum* were enumerated on a solid overlay medium containing ferrous sulfate (iFeo; Johnson and Hallberg 2007). Culture liquors were diluted and 100 µl aliquots spread onto solid media, which were incubated at 40 °C and 100% humidity for up to 10 days. Colonies of *L. ferriphilum* were identified by their deposition of ferric iron and rust-like colorations.

## Results and discussion

### Growth of pure and mixed cultures of *L. ferriphilum* and *A. caldus*

**Fig. 1:**
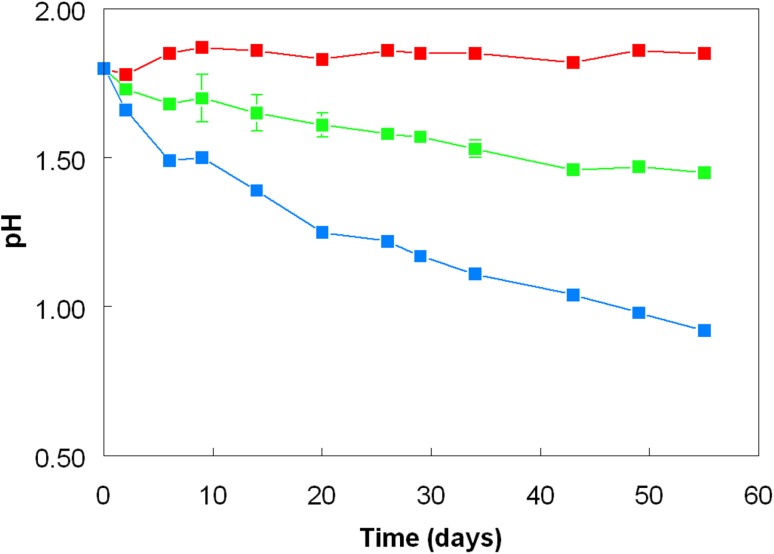
Changes in pH in pure and mixed cultures of *A. caldus* and *L. ferriphilum* grown in the presence of ferrous or ferric iron, with or without zero-valent sulfur (ZVS). Key: (filled blue square) *A. caldus,* Fe(III)/ZVS; (filled red square) *A. caldus*/*L. ferriphilum*, Fe(II); (filled green square) *A. caldus*/*L. ferriphilum*, Fe(II), ZVS. Symbols depict mean values and error bars (where visible) standard deviations

The pH trends observed in experiment 1 differed between cultures (Fig. 1). In the case of the mixed culture grown in sulfur-free medium, pH increased marginally as a result of ferrous iron oxidation (Fe^2+^ + 0.25 O_2_ + H_3_O^+^ → Fe^3+^ + 1.5 H_2_O) while in the sulfur/ferric iron control containing only *A. caldus* the pH declined throughout the time course of the experiment, reaching pH 0.92 (equivalent to an hydronium ion concentration of 120 mM) at day 55 as a consequence of microbiological oxidation of elemental sulfur and production of sulfuric acid (S^0^ + 1.5 O_2_ + 2 H_2_O → H_3_O^+^ + HSO_4_^−^). The pH also declined in the mixed cultures of *A. caldus and L. ferriphilum* in sulfur/ferrous iron medium, but far more slowly than in the pure *A. caldus* control culture, and was pH 1.45 (equivalent to an hydronium ion concentration of 35 mM) at day 55. The fact that mixed cultures containing initially both ferrous iron and elemental sulfur were net productive of acid can be explained by their far greater potential for hydronium ion production (156 mM, for 100% sulfur oxidation) than hydronium ion consumption (5 mM).

**Fig. 2:**
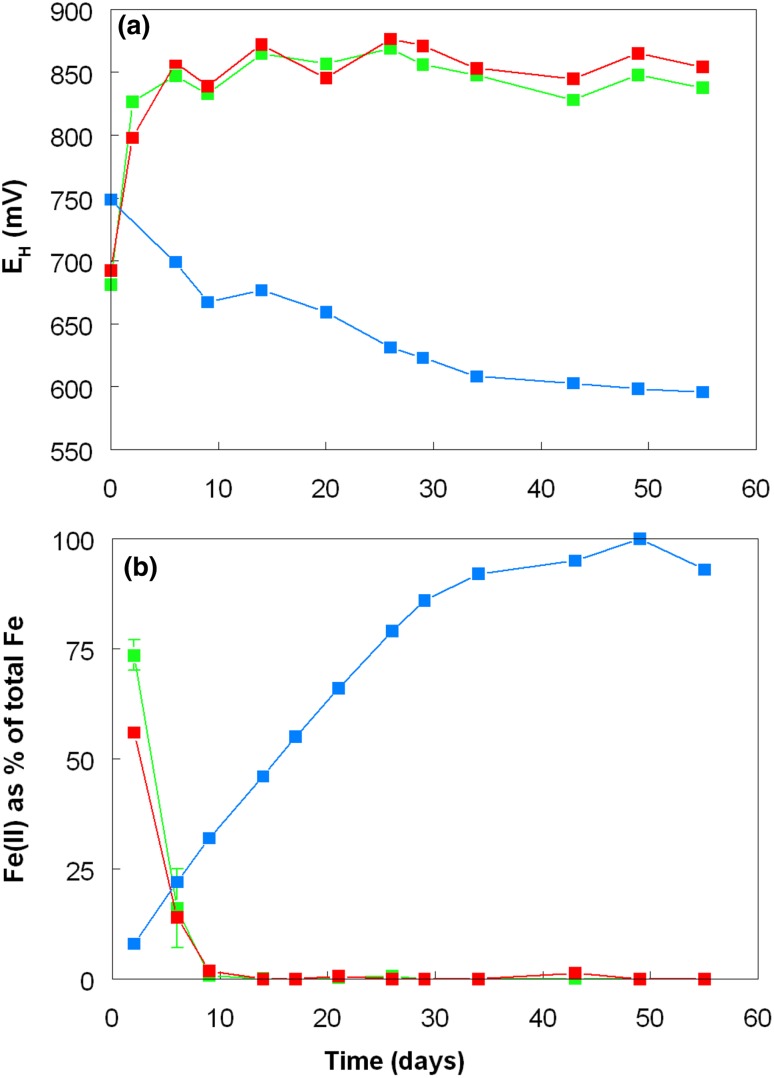
Changes in **a** redox potentials and **b** relative proportions of ferrous and ferric iron in pure and mixed cultures of *A. caldus* and *L. ferriphilum* grown in the presence of ferrous or ferric iron, with or without zero-valent sulfur (ZVS). Key: (filled blue square) *A. caldus,* Fe(III)/ZVS; (filled red square) *A. caldus*/*L. ferriphilum*, Fe(II); (filled green square) *A. caldus*/*L. ferriphilum*, Fe(II), ZVS. Symbols depict mean values and error bars (where visible) standard deviations

The redox potentials in the cultures reflected the relative concentrations of ferrous and ferric iron present at different times (Fig. 2). In all cultures that contained *L. ferriphilum*, ferrous iron was oxidized rapidly and *E*_H_ values increased to > + 850 mV, and remained as such for the duration of the experiment. In contrast, ferrous iron concentrations increased progressively (and *E*_H_ values became less positive) with time in the pure culture of *A. caldus*, in agreement with the previous report of Johnson et al. (2017). In acidic aerobic solutions (in the absence of an iron-oxidizing acidophile) ferrous iron is stable, and not prone to spontaneous re-oxidation as in higher pH liquors (Johnson et al. 2012).

**Fig. 3:**
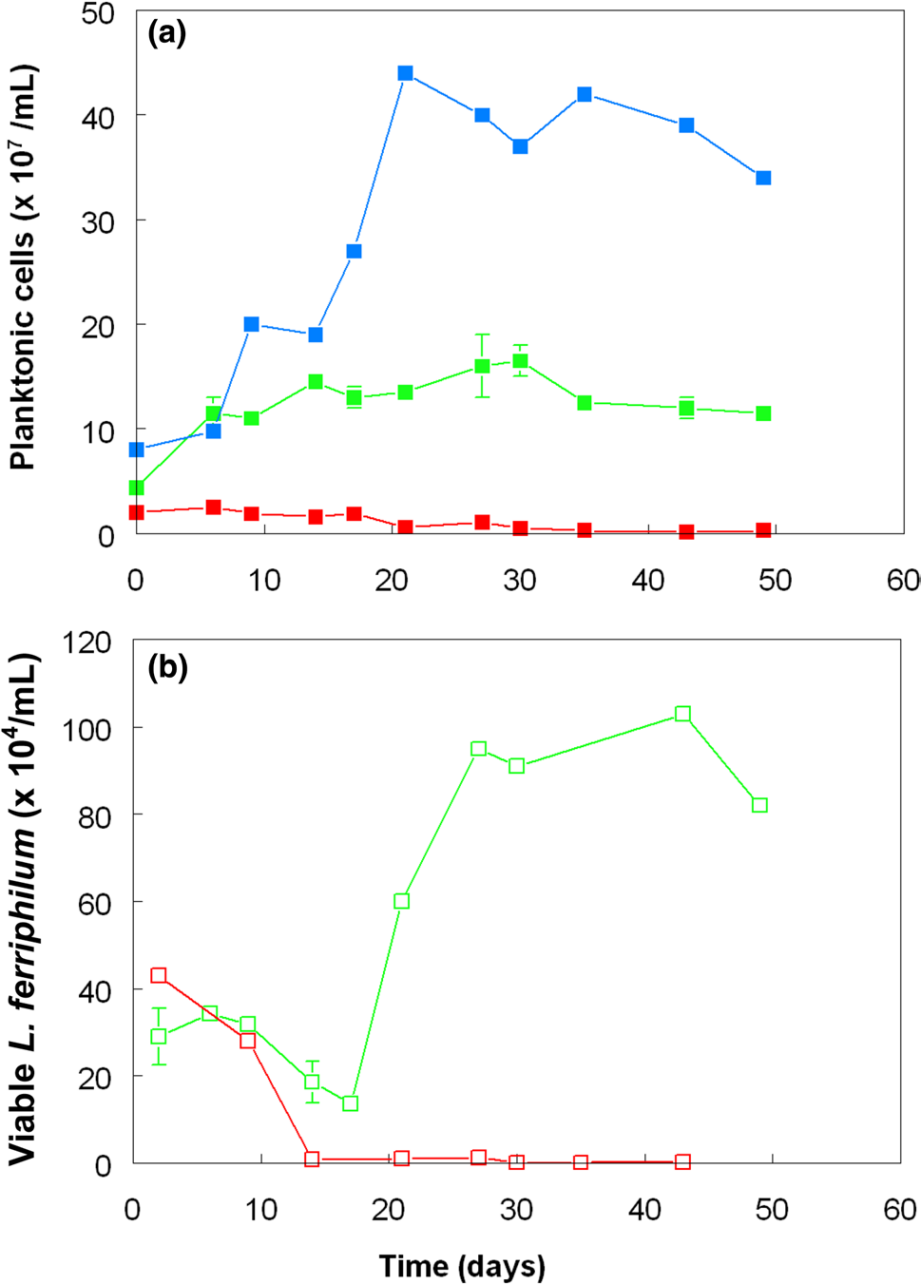
Changes in **a** total numbers of planktonic bacteria and **b** viable *L. ferriphilum* in pure and mixed cultures of *A. caldus* and *L. ferriphilum* grown in the presence of ferrous or ferric iron, with or without zero-valent sulfur (ZVS). Key: (filled blue square) *A. caldus,* Fe(III)/ZVS; (filled red square/empty red square) *A. caldus*/*L. ferriphilum*, Fe(II); (filled green square/empty green square) *A. caldus*/*L. ferriphilum*, Fe(II), ZVS. Symbols depict mean values and error bars (where visible) standard deviations

Phase contrast observations of the mixed cultures confirmed that most planktonic cells present were straight rods, corresponding to *A. caldus,* rather than curved rods typical of *L. ferriphilum*. This conclusion was also supported by data from the control cultures, where numbers of *A. caldus* (pure culture control) were over two orders of magnitude greater than those in the sulfur-free control, where only *L. ferriphilum* could grow. Interestingly, numbers of planktonic *A. caldus* in the pure culture control were, after day 6 of the experiment, far greater than those in the mixed cultures (which were predominantly *A. caldus*; Fig. 3a), and paralleled the trends observed with acid generation (Fig. 1). Numbers of viable planktonic *L. ferriphilum* declined rapidly after all of the ferrous iron had been oxidized in the sulfur-free control culture (Fig. 3b). A similar scenario was previously reported for the closely related iron-oxidizing acidophile *L. ferrooxidans*, which was found to lose viability far more rapidly than another iron-oxidizing chemolithotroph (*A. ferrooxidans*) once all of the ferrous iron provided in batch cultures had been oxidized (Johnson et al. 2004). In contrast, in cultures containing elemental sulfur as well as iron, numbers of viable planktonic *L. ferriphilum* declined between days 6 and 17, but after that increased fourfold (to ~ 10^6^/mL) and were sustained at similar numbers for the duration of the experiment. *Leptospirillum* spp. have high affinities for ferrous iron (Norris et al. 1988) which allows them to use this, their sole known electron donor, when present in very low (micro-molar) concentrations. The perpetual high positive *E*_H_ values and low ferrous iron concentrations found in these cultures implies that iron cycling was in a state of dynamic equilibrium, being continuously generated (indirectly) by *A. caldus* (as shown in the pure culture control, and in previous work) and re-oxidized by *L. ferriphilum*. The hypothesis that iron-oxidizing *L. ferriphilum* can grow in aerobic sulfur-containing media in co-culture with *A. caldus* was therefore confirmed.

### Inhibition of growth of *A. caldus* in mixed cultures containing *L. ferriphilum*

**Fig. 4:**
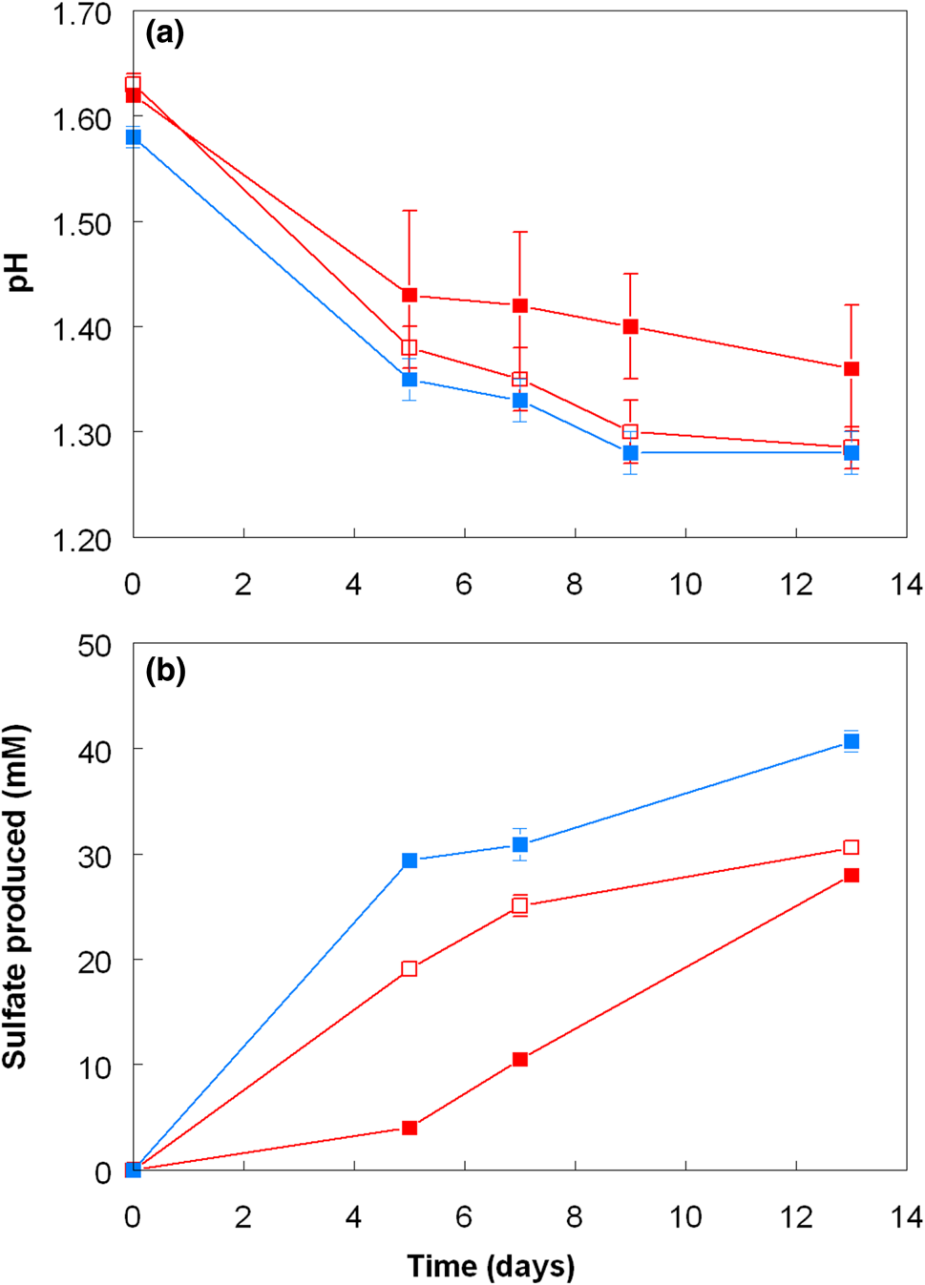
Changes in **a** pH and **b** sulfate production in cultures of *A. caldus* grown on zero-valent sulfur in spent (oxidized) culture medium of *L. ferriphilum* containing cells of the iron-oxidizer (filled red square) and medium with cells removed by centrifugation and filtration (empty red square). Data from cultures grown in a synthetic medium containing equivalent amounts of ferric iron are shown as filled blue square. Symbols depict mean values and error bars (where visible) standard deviations

**Fig. 5:**
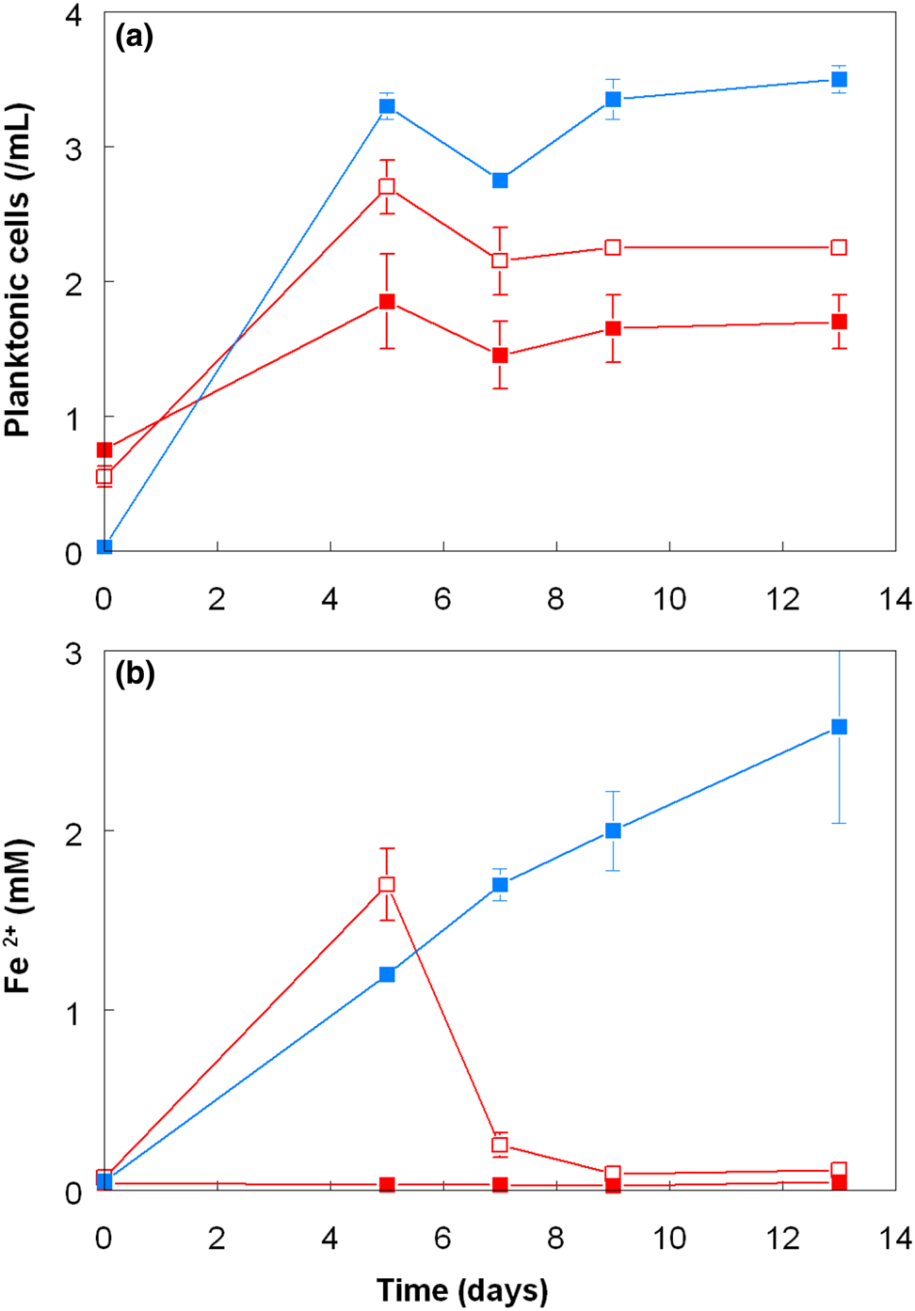
Changes in **a** numbers of planktonic cells and **b** ferrous iron concentrations in cultures of *A. caldus* grown on zero-valent sulfur in spent (oxidized) culture medium of *L. ferriphilum* containing cells of the iron-oxidizer (filled red square) and medium with cells removed by centrifugation and filtration (empty red square). Data from cultures grown in a synthetic medium containing equivalent amounts of ferric iron are shown as filled blue square. Symbols depict mean values and error bars (where visible) standard deviations

Experiment 1 produced data that were not anticipated, namely that growth and sulfur oxidation by *A. caldus* appeared to be partially inhibited in the presence of *L. ferriphilum*. To investigate this further, experiment 2 was set up, as described above. The pH, *E*_H_ and cell numbers in the fully oxidized iron-grown *L. ferriphilum* culture were 1.56, + 903 mV, and 6.3 × 10^7^/mL, respectively. Data comparing the performance of *A. caldus* in this oxidized culture medium (with and without *L. ferriphilum* cells) to that in a liquid medium with essentially the same chemical composition but prepared abiotically, are shown in Figs. 4, 5. In general, similar trends were observed as in the first experiment, though differences between cultures were more cryptic, due in part to the shorter duration of experiment 2 (13 days) than experiment 1 (55 days). The pH in cultures of *A. caldus* in the synthetic medium and “cell-free” spent culture medium declined at similar and faster rates than in spent cultures medium that contained *L. ferriphilum* cells (Fig. 4a). Mean increases in sulfate concentrations were also faster in synthetic than in spent medium, though these were marginal and sometimes masked by variations between replicate cultures (Fig. 4b). As in the first experiment, numbers of planktonic *A. caldus* cells were greater in pure cultures than in those where *L. ferriphilum* was also present; numbers in the “cell free” spent culture medium were intermediate between the two (Fig. 5a). Ferrous iron concentrations in *A. caldus* grown in synthetic media increased throughout experiment 2 whereas, as anticipated, they remained close to detection limits in the presence of *L. ferriphilum* (Fig. 5b). In oxidized media that had been manipulated to remove *L. ferriphilum*, ferrous iron concentrations also increased up to day 5, but thereafter decreased to very low levels. Examination confirmed the presence of viable *L. ferriphilum* in both replicate cultures, and that the protocol used for completely removing these bacteria from oxidized cultures had not been totally successful, though the short-term results (i.e. before significant proliferation of *L. ferriphilum*) were similar to those found in the first experiment.

### Effect of iron speciation on growth and activity of *A. caldus*

**Fig. 6:**
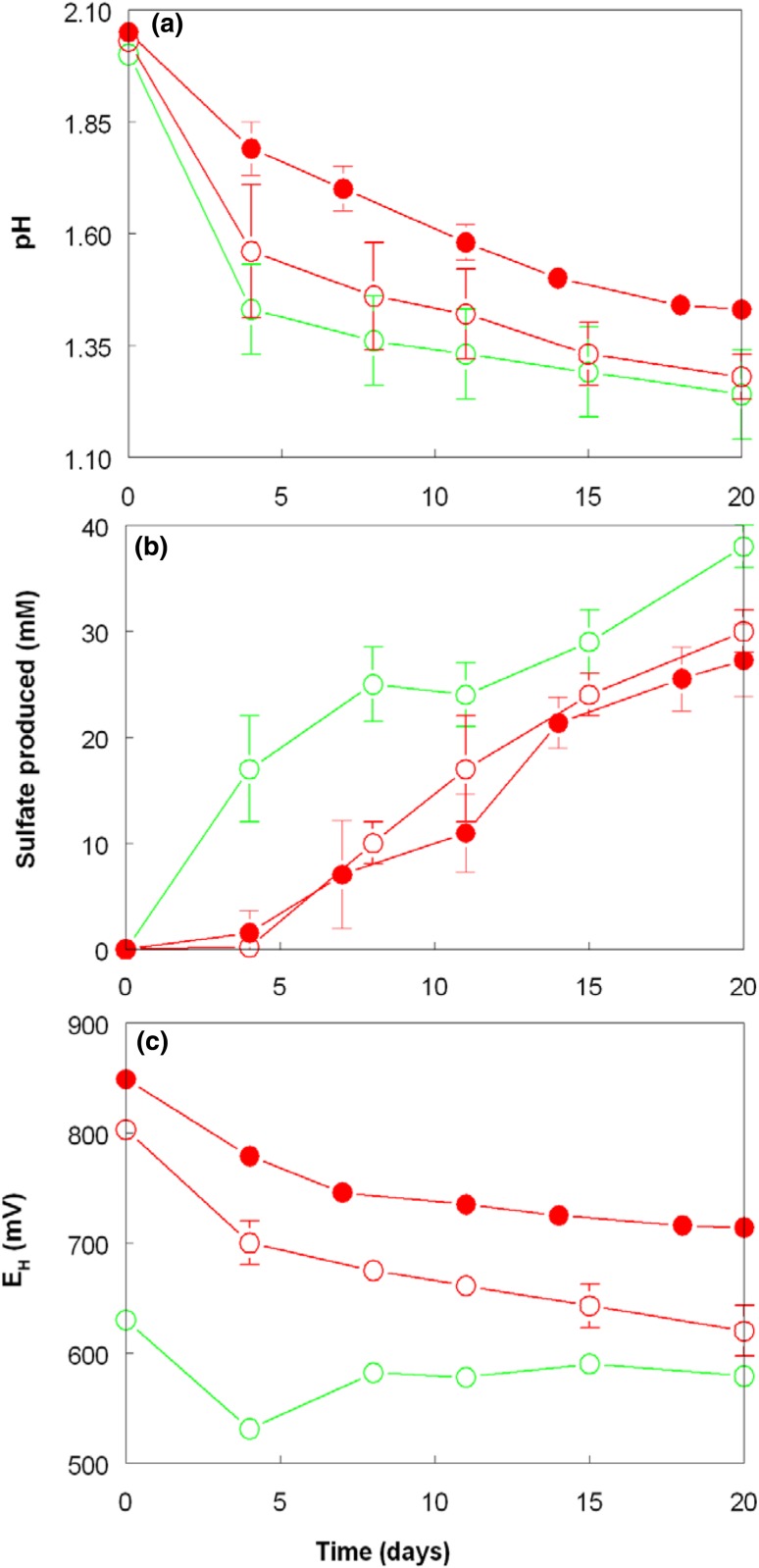
Changes in **a** pH, **b** sulfate production and **c** redox potentials in cultures of *A. caldus* grown on zero-valent sulfur in liquid media containing 5 mM ferrous iron (empty green circle), 5 mM ferric iron (empty red circle) or 25 mM ferric iron (filled red circle). Symbols depict mean values and error bars (where visible) standard deviations

Various reasons for the apparent inhibition of *A. caldus* when grown in co-culture with *L. ferriphilum* were considered. Both are autotrophic, and therefore compete for available CO_2_ which is poorly soluble in acidic liquors. However, since numbers of *L. ferriphilum* were about two orders of magnitude less than those of *A. caldus*, competition for CO_2_ was not considered to be significant, as were concentrations of macro-and micro-nutrients, which were present well in excess of those required for growth of both bacteria. Another possible explanation was a differential sensitivity of *A. caldus* to ferrous and ferric iron. In the presence of *L. ferriphilum* (re)-oxidation of ferrous iron generates high redox potential, ferric iron-dominated liquors, whereas ferric iron is progressively reduced to ferrous in axenic cultures of *A. caldus* grown aerobically. Results from experiment 3 showed that both pH declined and concentrations of sulfate increased more rapidly when ferrous iron rather than ferric iron was added to pure cultures of *A. caldus* (Fig. 6a, b) though final pH values were similar, possibly as a result of reduction of ferric iron in those cultures which contained this form of iron (dissimilatory reduction of soluble ferric iron is a proton-generating reaction; Johnson et al. 2012). Again, lowering of *E*_H_ values with length of incubation confirmed that ferric iron reduction was an ongoing process in aerobic cultures of *A. caldus* (Fig. 6c). Numbers of planktonic cells in ferrous iron cultures (5.65 ± 0.05 × 10^8^/mL) were also greater at day 20 than in those containing either 5 mM ferric iron (4.2 ± 1.3 × 10^8^/mL) or 25 mM ferric iron (2.85 ± 0.05 × 10^8^/mL). These results indicate that ferric iron sensitivity and highly positive redox potentials were responsible for the slower rates of both sulfur oxidation and growth of *A. caldus* when it was grown aerobically in mixed culture with *L. ferriphilum*, rather than in pure culture. Ferric iron has often been reported to be more toxic to acidophilic bacteria than ferrous iron (e.g. Falagán and Johnson 2016). Reduction of ferric iron by aerobically growing cultures of *A. caldus* can therefore be considered as a self-protection mechanism, though this only is effective where iron-oxidizing acidophiles, which inhabit similar environments to *A. caldus*, are inactive (e.g. as a result of extremely low pH).

### Environmental and industrial implications

Data from these experiments are significant in both environmental and applied contexts. By scavenging ferrous iron generated by sulfur-oxidizing *Acidithiobacillus* spp. in solfatara and other low pH sulfur-rich environments, *Leptospirillum* spp. would be able to survive and grow even in situations where concentrations of soluble iron are very small. Both *A. caldus* and *L. ferriphilum* are also two of the most important and abundant bacteria in commercial biomining operations (bio-heaps and especially stirred tanks; Rawlings and Johnson 2007) both of which require aerobic conditions. Cryptic reduction of ferric iron by *A. caldus* is, in theory, counter-productive to the objective of optimizing sulfide mineral dissolution (which is mediated by ferric iron) though the highly positive *E*_H_ values of most bioleach liquors implies that this is counter-balanced by re-oxidation of ferrous iron by *L. ferriphilum* when biomining systems are operating effectively. It has also been suggested that reductive bio-processing of lateritic ores could also be carried out under aerobic conditions. Marrero et al. (2015) showed that re-oxidation of ferrous iron did not occur in laterite-containing cultures that had an initial pH 0.8 and contained only *Acidithiobacillus* spp., presumably because these extremely low pH values inhibit the growth of (or kill) iron-oxidizing acidithiobacilli (Sand 1989; Johnson et al. 2017). However, *Leptospirillum* spp. can grow at much lower pH values (> pH 0.5), the implication being that high redox potentials would be maintained in the presence of these acidophilic iron-oxidizers. Whilst axenic cultures of *A. caldus* could, in theory, be effective in reductive mineral bio-processing operations as they generate both acidity and low redox environments, since biomining necessarily operates in non-sterile conditions it might be difficult, if not impossible, to exclude *L. ferriphilum,* which has similar pH and temperature ranges as *A. caldus*. Ferrous iron would be regenerated, and highly positive redox potentials maintained when *L. ferriphilum* was present, precluding the reductive dissolution of goethite and other oxidized minerals present in lateritic ores.
